# Transcriptome Analysis Reveals the Mechanism Underlying the Production of a High Quantity of Chlorogenic Acid in Young Leaves of *Lonicera macranthoides* Hand.-Mazz

**DOI:** 10.1371/journal.pone.0137212

**Published:** 2015-09-18

**Authors:** Zexiong Chen, Ning Tang, Yuming You, Jianbin Lan, Yiqing Liu, Zhengguo Li

**Affiliations:** 1 Genetic Engineering Research Center, School of Life Sciences, Chongqing University, Chongqing, 400044, China; 2 College of Forestry and Life Science, Chongqing University of Arts and Sciences, Chongqing, 402160, China; Huazhong university of Science and Technology, CHINA

## Abstract

*Lonicera macranthoides* Hand.-Mazz (*L*. *macranthoides*) is a medicinal herb that is widely distributed in southern China. The biosynthetic and metabolic pathways for a core secondary metabolite in *L*. *macranthoides*, chlorogenic acid (CGA), have been elucidated in many species. However, the mechanisms of CGA biosynthesis and the related gene regulatory network in *L*. *macranthoides* are still not well understood. In this study, CGA content was quantified by high performance liquid chromatography (HPLC), and CGA levels differed significantly among three tissues; specifically, the CGA content in young leaves (YL) was greater than that in young stems (YS), which was greater than that in mature flowers (MF). Transcriptome analysis of *L*. *macranthoides* yielded a total of 53,533,014 clean reads (average length 90 bp) and 76,453 unigenes (average length 703 bp). A total of 3,767 unigenes were involved in biosynthesis pathways of secondary metabolites. Of these unigenes, 80 were possibly related to CGA biosynthesis. Furthermore, differentially expressed genes (DEGs) were screened in different tissues including YL, MF and YS. In these tissues, 24 DEGs were found to be associated with CGA biosynthesis, including six phenylalanine ammonia lyase (*PAL*) genes, six 4-coumarate coenzyme A ligase (*4CL*) genes, four cinnamate 4-Hydroxylase (*C4H*) genes, seven hydroxycinnamoyl transferase/hydroxycinnamoyl-CoA quinate transferase *HCT/HQT* genes and one coumarate 3-hydroxylase (*C3H*) gene.These results further the understanding of CGA biosynthesis and the related regulatory network in *L*. *macranthoides*.

## Introduction

*Lonicera*. *macranthoides* (*L*. *macranthoides*), a plant distributed widely in south China, belongs to Caprifoliaceae and is often used in traditional Chinese medicine. In the 2010 *Chinese Pharmacopoeia*, *L*. *macranthoides* is registered as “mountain honeysuckle” [1]. In light of the preventive and therapeutic role of *L*. *macranthoides* in the severe acute respiratory syndrome (SARS, 2003) and H1N1 (2009) outbreaks in China, the demand for *L*. *macranthoides* has dramatically increased. The entire plant of *L*. *macranthoides*, and particularly the buds and leaves, is used as medicine. Phenolic acids, flavonoids, volatile oils and saponins are major chemical components of *L*. *macranthoides* [2]. These components predominately account for the multiple medicinal effects of *L*. *macranthoides*, which include antioxidant [3,4,5], detoxification [6], anti-inflammatory [7], anti-cancer [8,9,10,11], anti-cardiovascular disease and pain relief properties [12]. Moreover, *L*. *macranthoides* can be effectively applied in the treatment of H1N1 respiratory syndrome and hand-foot-and-mouth disease [13]. Chlorogenic acid (CGA) is the most interesting of the biologically active ingredients of *L*. *macranthoides*.

CGA is a potent phenolic acids that is considered to have many important biological activities [14,15,16,17]. CGA is a group of esters created from certain trans-cinnamic acids, such as caffeic acid, ferulic acid and quinic acid [18,19]. Thirty different types of CGA have been identified in plants [19]. CGA is an important class of dietary antioxidants in a variety of fruits and vegetables, including apples, pears, tomatoes and potatoes [20] as well as other members of *Asteraceae*, *Solanaceae* and *Rubiaceae* [21]. CGA accumulates in the flowers, stems and leaves of *Lonicera* [22,23].

CGA is a phenylpropanoid generated from the shikimic acid pathway of plant aerobic respiration. *PAL*, *C4H* and *4CL* are key enzymes involved in the first three steps of CGA biosynthesis; however, the specific details of the roles of these enzymes in this process have not yet been fully elucidated [24]. Hydroxycinnamoyl-CoA quinate transferase (*HQT*) is a key enzyme acting downstream of the CGA metabolic pathway [25]. The rate-limiting role of HQT in CGA biosynthesis in tomato, coffee, artichoke and other plants [24] has been confirmed by *HQT* transgenic plants in tomato [20]. Moreover, the *HQT* gene may be indispensable in CGA synthesis in *L*. *japonica* [26]. Three different CGA synthesis pathways have been hypothesized. First, CGA is generated from caffeic acid coenzyme A and quinic acid via catalysis by HQT [20]. Second, CGA is produced from coumaroyl quinic acid mediated by hydroxycinnamoyl CoA shikimate/quinate hydroxycinnamoyl transferase (HCT) [27]. Third, caffeic acid glucosidase serves as an active intermediate [28]. We hypothesized that the key CGA biosynthetic pathways may be different in different species.

There have been reports on the molecular mechanisms of CGA biosynthesis in *Lonicera* plants. Peng *et al*. (2010) cloned the *HQT* gene in *L*. *japonica* and demonstrated a correlation between *HQT* expression and CGA content in different tissues [26]. Yuan et al. (2014) identified sequences of genes related to CGA synthesis including *PAL*, *4CL*, *C4H* and *HQT* using a transcriptome analysis in *L*. *japonica*, *L*. *hypoglauca* Miq. and *L*. *macranthoides* [13]. Several studies have performed transcriptome profiling in *L*. *japonica* in recent years. Genes involved in the biosynthesis of the active ingredients in *L*. *japonica* were screened by transcriptome analysis of the flower and leaf tissues using 454 pyrosequencing [29]. Yuan *et al*. (2012) identified genes associated with CGA and Luteoloside biosynthesis from transcriptome data in *L*. *japonica* [30]. However, there is no transcriptome-wide analysis of *L*. *macranthoides*, and the molecular mechanisms underlying the biosynthesis of important active components remain a mystery.

In the present study, CGA in different tissues were determined using high performance liquid chromatography (HPLC), and transcriptome-wide sequencing in *L*. *macranthoides* was performed using the Illumina HiSeq™ 2000 platform. To explore the correlation between key genes associated with the biosynthesis and metabolism of CGA and CGA contents, digital gene expression (DGE) analysis was performed on different tissues including flower, stem and leaf. RNA-Seq analysis identified differentially expressed genes (DEGs) that are potentially important for secondary metabolism and CGA biosynthesis in *L*. *macranthoides*. These results are practical genomic resources for future investigations into the biosynthesis of CGA and for potential genetic improvement of *L*. *macranthoides*.

## Materials and Methods

### Plant Materials

Plant materials including mature flowers (MF, 5–6 cm in length and collected at 1 day after anthesis in color of light yellow), young leaves (YL, 10-days old) and young stem (YS, 10-days old) were collected for transcriptome analysis and CGA content measurement. These samples were planted in the *L*. *macranthoides* test site in Chongqing China (the specific location was around N28.47, E108.97). All of the tissues were cut into small pieces, frozen in liquid nitrogen and stored at -80°C until further use.

### Determination of CGA Content by HPLC

Tissue samples were processed in a series of steps, including lyophilization, grinding and passage through a 40 mesh sieve before CGA extraction. Then, 50 mL of ethanol (70%, v/v) was mixed with 0.5 g of the processed samples, and the mixture was subjected to ultrasonic extraction for 30 min. It was then centrifuged at 4000 rpm for 10 min. The supernatant was filtered through 0.45 μM microfiltration membrane for CGA analysis.

HPLC was performed on a Shimadzu LC-20A HPLC analyzer (Shimadzu Corporation, Kyoto, Japan), equipped with a LC-20AT pump, SIL-20A autosampler, CBM-20A system controller, SPD-M20A diode array detector and CTO-20A column oven. Separation was carried out using a Shimadzu Shim-PackVP-ODS C18 column (5 μm, 250×4.6 mm) and 2% formic acid (in methanol, 80:20, V/V) as the mobile phase at a 1.0 mL/min flow rate. The column and the detector were operated at 35°C. A volume of 20 μL was injected and the HPLC chromatogram was monitored at 320 nm. CGA standard (≥ 98%) was purchased from SIGMA (Sigma-Aldrich, St. Louis, MO, USA). The CGA contents were analyzed in triplicate and calculated based on peak area measurements. Statistical significance was performed with SPSS using Duncan's new multiple range test.

### Transcriptome Analysis

Total RNA was extracted from the tissues, including flower, leaf and stem, using TRIzol reagent (Invitrogen, USA). Equal amounts of RNA from each of the three, tissues were mixed for transcriptome sequencing. Poly A mRNA was enriched using oligo (dT) magnetic beads and fragmented. The short fragments were served as templates to synthesize first strand cDNA using random primers, and then the second cDNA strand was synthesized. Subsequently, cDNAs were end-repaired, A-tailed and ligated with sequencing adaptors. The cDNAs were size selected and PCR amplified to generate the final library for sequencing using the Illumina HiSeq™ 2000 platform.

### De Novo Transcriptome Assembly

The raw reads were generated via image analysis and base calling procedures. Then, the raw reads were filtered before assembly and mapping by removing adaptors, reads with an N ratio greater than 5% and low-quality reads, which resulted in clean reads. Trinity software [31] was employed for *de novo* transcriptome assembly.

First, overlapping reads of a certain length were assembled into contigs. Then, the paired-end reads were mapped to contigs, and the different contigs in the same transcript were identified, as well as the distances between them. Subsequently, contigs were assembled using Trinity to generate a unigene sequence that could not be extended at either of the two ends. The final set of unigenes was obtained using homologous clustering of the assembled transcripts and by removing redundant unigenes using TGICL (TGI clustering tools) [32].

After homologous clustering, the unigenes were divided into two categories, clusters and singletons. In the same cluster, there were several unigenes with relatively high similarity (greater than 70%), which are noted as ‘CL’ followed by the gene family ID. The singletons represent single unigenes and are noted as ‘Unigene’ followed by the gene ID. Finally, the unigenes were aligned to protein sequence database entries including those in the NCBI non-redundant (Nr), Swiss-Prot, Kyoto Encyclopedia of Genes and Genomes (KEGG) and Clusters of Orthologous Groups of proteins (COG) databases using BLASTx (e-value < 0.00001). The best alignment result was used to determine the direction of the unigene sequence. If the above four databases were not available, ESTScan software was employed to determine the direction of the sequence [33].

### Functional Annotation, Gene Ontology (GO) Classification and Analysis of Metabolic Pathway

To obtain functional annotation of a given unigene, the sequence was aligned against protein sequence database entries including those in Nr, Swiss-Prot, KEGG and COG using BLASTx, and aligned against nucleotide sequence database using BLASTn with a significance threshold of e-value < 0.00001. Based on Nr annotation, the GO categories were obtained using Blast2GO software [34]. Then, GO classifications for all unigenes were visualized using WEGO software [35]. Pathway analysis was performed with Blastall software using the KEGG database.

### DGE Profiling Analysis

Total RNA was extracted from different tissues including YL, YS and MF. Three libraries were established for RNA-Seq analysis, which were then sequenced using the Illumina HiSeq™ 2000 platform.

The raw image data obtained from the sequencing were transformed into raw reads with base calling. Poor quality reads were removed to obtain clean reads for subsequent analyses. Short read alignment software SOAP aligner/soap2 [36] was used to align the clean reads to the reference gene sequences (allowing two-base mismatch). The obtained sequences were evaluated using the distribution of reads in the reference genes and sequence saturation analysis.

Gene expression was calculated using the number of reads aligned to a single gene and the total number of reads aligned to reference sequences in the Reads per Kb per Million reads (RPKM) method [37], and the gene information was specified for coverage and functional annotation.

DEGs were identified by comparing data obtained from different samples as described by Audic et al. [38]. In multiple testing and analysis, false discovery rate (FDR) was used to determine the p-value threshold. In this study, "FDR≤0.001 and the value of log2 Ratio≥1" was set as the threshold to judge the significance of gene expression difference.

With Cluster software [39], Euclidean distance was used in the distance matrix for simultaneous hierarchical clustering analyses of DEGs, and the clustering result was displayed using Java Treeview [40]. For GO enrichment analysis, all DEGs were mapped to different terms in the GO database (http://www.geneontology.org/), and the number of genes mapped to each term was noted. Next, hypergeometric test were used to determine GO terms that were significantly enriched in the DEGs compared with the entire genome background. For pathway enrichment analysis, all DEGs were mapped to the KEGG database, and pathways with Q value ≤ 0.05 were considered significantly enriched in DEGs.

### Validation of RNA-Seq Data by Quantitative Real-Time PCR (qRT-PCR)

To validate the accuracy of the gene expression levels of DEGs obtained from the RNA-Seq analysis, six DEGs from the three tissues and five genes possibly associated with CGA synthesis were randomly selected and subjected to qPCR detection. Gene-specific primers for selected genes were designed using online primer design software (https://www.genscript.com/ssl-bin/app/primer) (S1 Table) and a melting curve analysis was used to confirm specificity. qRT-PCR were performed using a Fast SYBR Mixture (CWBIO, Beijing) on an Bio-Rad CFX connect real-time PCR detection system using of 95°C incubation for 10 min, then 40 cycles of 95°C for 15 s and 60°C for 60 s. For all qPCR experiments, three biological replicates were performed. Relative expression levels were calculated based on the 2^-ΔΔCt^ method using *tubulin* as a reference gene.

### Sequence Deposition

The raw transcriptome reads reported here have been deposited in the NCBI Short Read Archive under accession Nos. SRX1003341, and the raw DGE data were deposited under accession Nos. SRX1003347 (MF), SRX1003358 (YL) and SRX1003352 (YS).

### Ethics Statement

Experiment was carried out in the field own by College of Forestry and Life Science, and no specific permissions were required for these locations/activities. No endangered or protected species or locations were involved in this study.

## Results

### CGA Contents in Different *L*. *macranthoides* Tissues

**Fig 1:**
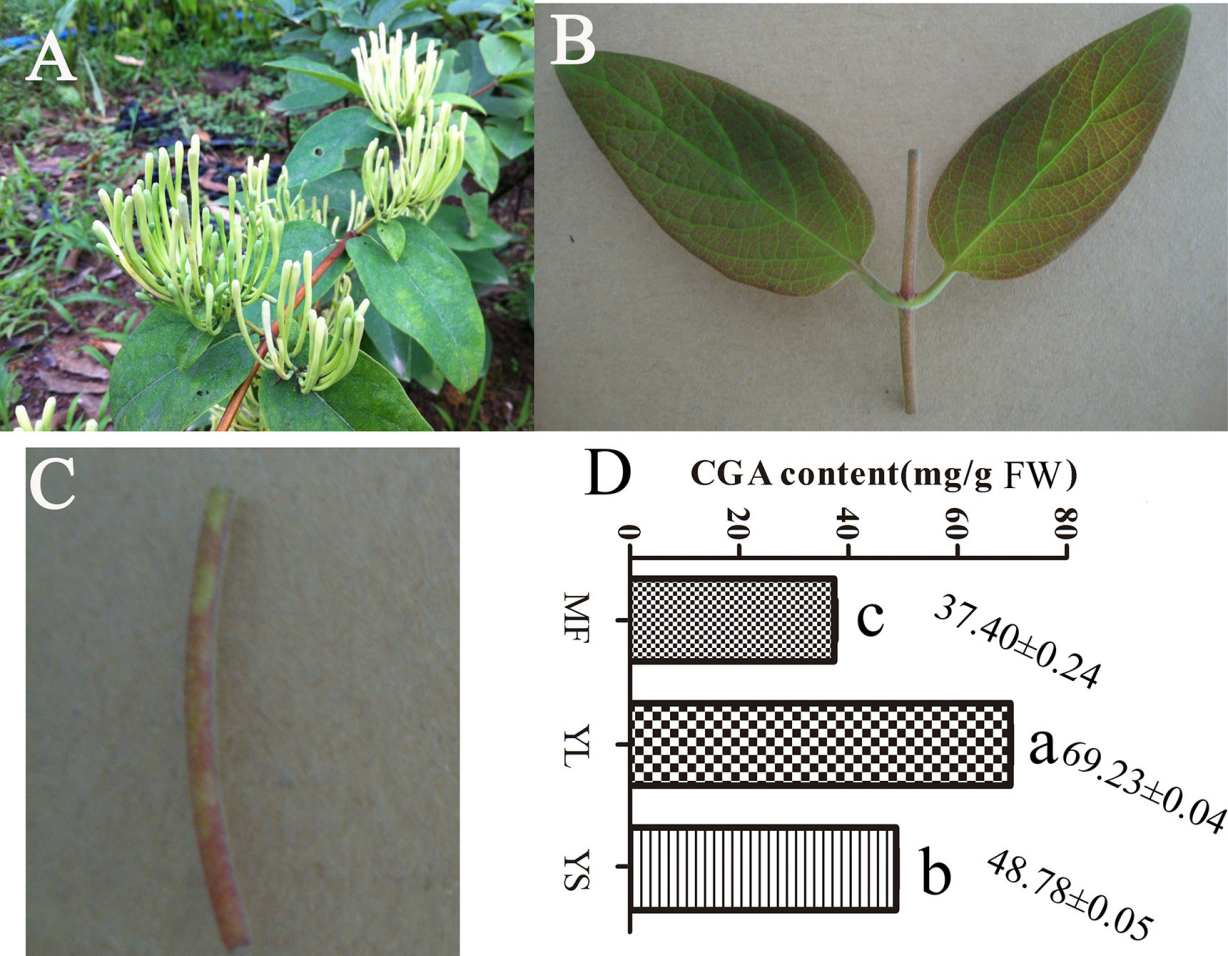
Tissues of *Lonicera macranthoides* used in deep sequencing and CGA content in each detected tissue. A, B and C were mature flower, young leaf and young stem of *Lonicera macranthoides*, D was the CGA content in fresh MF, YL and YS using HPLC, three individual experimental were performed for each tissue, and significant differences were analysed using student’s t test, different alphabet indicated *P* value<0.05.

The CGA contents in the MF, YL and YS of *L*. *macranthoides* were measured using HPLC (Fig 1D). The CGA content differed significantly between the three tissues (*P* < 0.05), YL (69.7333 ± 0.0378, Mean ± SE mg/g FW) > YS (48.7857 ± 0.0523) > MF (37.4027 ± 0.2437).

### Illumina Sequencing and *de novo* Assembly

Because genomic sequences of *L*. *macranthoides* were not available, transcriptome analysis of mixed tissues was performed to obtain genome data. A total of 60,287,590 raw reads were obtained, corresponding to 53,533,014 clean reads with an average length of 90 bp. These reads were further assembled into 145,214 sequences (contigs), with an average length of 342 bp (S2 Table). After processing by Trinity and TGICL software, 145,214 contigs were further assembled into 76,453 unigenes with an average length of 703 bp and an N50 length of 1088 bp (i.e., half of the assembled bases were incorporated into unigenes with a length of at least 1088 bp, S2 Table). The specific size distribution of these sequences and non-repetitive genes is shown in S1 Fig

### Functional Annotation

Approximately 66.3% of the unigenes (50,701) were annotated using BLASTx, with a threshold of 10^−5^, considering four public databases, the nr database, the Swiss-Prot protein database, the KEGG database, and the COG database. Among them, 50561, 31450, 28077 and 17183 unigenes could be annotated in the Nr database, swissprot database, KEGG database and COG database, respectively.

The *L*. *macranthoides* unigenes were assigned three GO categories, molecular function, cellular component and biological process. Based on sequence homology, 38,533 sequences were classified into 47 functional groups of the three ontologies (S2 Fig). For the molecular function ontology category, cellular process and metabolic process were the most predominant, whereas subcategories such as carbon utilization, locomotion, nitrogen utilization, sulfur utilization and viral reproduction included relatively few members (S2 Fig). GO analysis showed that the identified genes are involved in various biological processes. In total, 24,735 sequences were annotated in the metabolic process category, suggesting that our study may have identified novel genes related to secondary metabolism pathways.

KEGG is a database for the systematic analysis of gene functions according to the metabolic pathways. A total of 28,044 unigenes in *L*. *macranthoides* were mapped to 128 KEGG pathways. Among them, the most represented pathways are metabolic pathways (6514), biosynthesis of secondary metabolites (3194) and plant-pathogen interaction (1633). These annotations provide valuable information for further investigations of the special metabolic processes, functions and pathways in *L*. *macranthoides*. Interestingly, 3767 unigenes were mapped to 33 pathways associated with the biosynthesis of secondary metabolites (Table 1). Among them, phenylpropanoid biosynthesis [PATH: Ko00940] is the largest family (447, 11.9%), followed by zeatin biosynthesis [Ko00908] (262, 7.0%), flavonoid biosynthesis [Ko00941] (249, 6.6%) and stilbenoid, diarylheptanoid and gingerol biosynthesis [Ko00945] (235, 6.2%). CGA is a type of phenylpropanoid produced in plants via the phenylpropanoid biosynthesis pathway during aerobic respiration and is a caffeoylquinic acid derivative. A total of 80 genes encoding five enzymes involved in CGA biosynthesis were identified by transcriptome analysis in *L*. *macranthoides* Hand.-Mazz. (Table 2). This data set is a crucial genetic resource for gene engineering studies on *L*. *macranthoides*.

**Table 1:** The unigenes related to secondary metabolites.

Biosynthesis of Secondary Metabolites	Unigene Numbers	Pathway ID
Anthocyanin biosynthesis	23	Ko00942
Ascorbate and aldarate metabolism	197	Ko00053
Betalain biosynthesis	2	Ko00965
Biotin metabolism	9	Ko00780
Brassinosteroid biosynthesis	89	Ko00905
Caffeine metabolism	8	Ko00232
Carotenoid biosynthesis	215	Ko00906
Cutin,suberine and wax biosynthesis	135	Ko00073
Diterpenoid biosynthesis	148	Ko00904
Flavone and flavonol biosynthesis	135	Ko00944
Flavonoid biosynthesis	249	Ko00941
Folate biosynthesis	46	Ko00790
Glucosinolate biosynthesis	29	Ko00966
Glycosaminoglycan degradation	88	Ko00531
Indole alkaloid biosynthesis	56	Ko00901
Isoflavonoid biosynthesis	60	Ko00943
Isoquinoline alkaloid biosynthesis	68	Ko00950
Limonene and pinene degradation	232	Ko00903
Monoterpenoid biosynthesis	30	Ko00902
Nicotinate and nicotinamide metabolism	30	Ko00760
Phenylalanine metabolism	186	ko00360
Phenylpropanoid biosynthesis	447	Ko00940
Porphyrin and chlorophyll metabolism	142	Ko00860
Riboflavin metabolism	49	Ko00740
Sesquiterpenoid and triterpenoid biosynthesis	58	Ko00909
Stilbenoid, diarylheptanoid and gingerol biosynthesis	235	Ko00945
Steroid biosynthesis	97	Ko00100
Thiamine metabolism	28	Ko00730
Tropane, piperidine and pyridine alkaloid biosynthesis	61	Ko00960
Terpenoid backbone biosynthesis	205	Ko00900
Ubiquinone and other terpenoid-quinone biosynthesis	108	Ko00130
Vitamin B6 metabolism	40	Ko00750
Zeatin biosynthesis	262	Ko00908
Total	3767	

**Table 2:** The numbers of Unigene involved in CGA biosynthesis.

Gene	Enzyme No.	Numbers
L-phenylalanin ammo-nialyase (PAL)	[EC:4.3.1.24]	10
4-Coumarate:CoA ligase(4CL)	[EC:6.2.1.12]	25
Cinnamate 4-hydroxylase(C4H)	[EC:1.14.13.11]	7
Hydroxycinnamoyl-CoA shikimate/quinate hydroxycinnamoyltransferase (HQT/HCT)	[EC: 2.3.1.133]	29
P-coumarate 3-hydroxylase(C3H)	[EC: 1.14.13.36]	9
Total	80	

### RNA-Seq Analysis

To further explore gene expression patterns in different tissues of *Lonicera macranthoides* Hand.-Mazz., three libraries including MF, YS and YL were established for RNA-Seq analysis. After filtering adaptor sequences and low quality sequences, the three RNA-Seq libraries generated at least 3.5 million clean reads (each) and the percentage of clean reads in each library was more than 99.4% (Fig A in S1 File).

The total reads of MF, YL and YS ranged from 3,550,236 to 3,720,194. After alignment, there were 3,071,878 (86.53%), 3,122,293 (86.72%) and 3,240,456 (87.10%) reads mapped to reference genes, respectively. Only reads aligning entirely inside exonic regions could be matched, whereas those from exon-exon junction regions could not be matched). For all the total mapped reads, the unique match and multi-position match in MF, YL and YS were 2,361,848 (66.53%) and 710,030 (20.00%), 2,438,931 (67.74%) and 683,362 (18.98%), and 2,495,081 (67.07%) and 745,375 (20.04%), respectively (Table 3).

**Table 3:** Summary of read numbers based on the RNA-Seq data from different tissues of *Lonicera macranthoides*.

	Mature Flower	Young Leaf	Young Stem
Total reads	3550236	3600370	3720194
Total base pairs	173961564	173961564	182289506
Total Mapped reads	3071878(86.53%)	3122293 (86.72%)	3240456(87.10%)
Perfect match	2599383(73.22%)	2598665(72.18%	2697153(72.50%)
Unique match	2361848(66.53%)	2438931(67.74%)	2495081(67.07%)
Multi-position match	710030(20.00%)	683362(18.98%	745375(20.04%)
Total unmapped reads	478358(13.47%)	478077(13.28%)	479738(12.90%)

### DEGs in Different Tissues

Gene coverage is the percentage of a gene covered by the reads. This value is equal to the ratio of the base number in a gene covered by unique mapping reads to the total bases number of that gene. The distribution of each tissue in the reference genome's coverage and sequencing depth are listed in are listed in S1 Fig The distribution of gene coverage in each region showed a similar pattern in all three RNA-Seq libraries (Fig B in S1 File). In each RNA-Seq library the gene coverage distribution proportion of different region ranged from 7% to15% and the number of genes ranged between 3724 and 7954 in each area.

**Fig 2:**
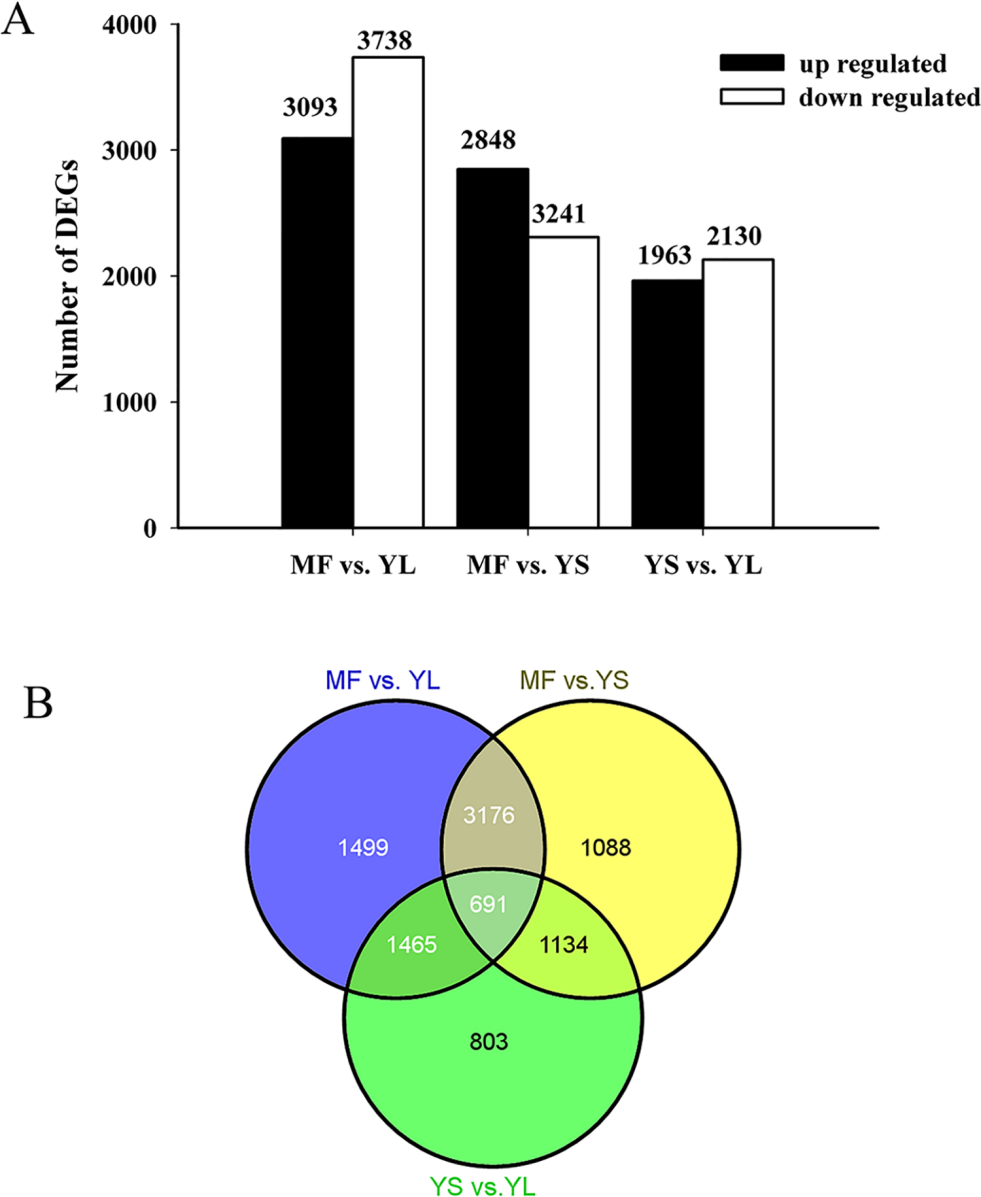
The numbers of DEGs between different tissues. Between the MF and YL libraries, there were 3093 up regulated genes and 3738 down regulated genes; Between the MF and YS libraries, there were 2848 up regulated genes and 3241 down regulated genes, while there were 1963 up regulated genes and 2130 down regulated genes between the YS and YL libraries. A total of 691 genes were differentially expressed among the MF, YL and YS libraries.

The differences in gene expression patterns were then analyzed for the pairs MF and YL, MF and YS, YS and YL (Fig 2A). A total of 691 genes were differentially expressed among the MF, YL and YS libraries (Fig 2B). A total of 6831 genes were differentially expressed between the MF and YL libraries (Fig 2A and Tables A-C in S2 File), among which 3093 were up-regulated and 3738 were down-regulated. Between the MF and YS libraries, 6089 genes were differentially expressed, including 2848 up-regulated and 3241 down-regulated genes (Fig 2A and Tables A-C in S2 File). The YS and YL libraries showed 4093 DEGs including 1963 up-regulated and 2130 down-regulated (Fig 2A and Tables A-C in S2 File).

### DEGs Associated with CGA Synthesis in Different Tissues

**Fig 3:**
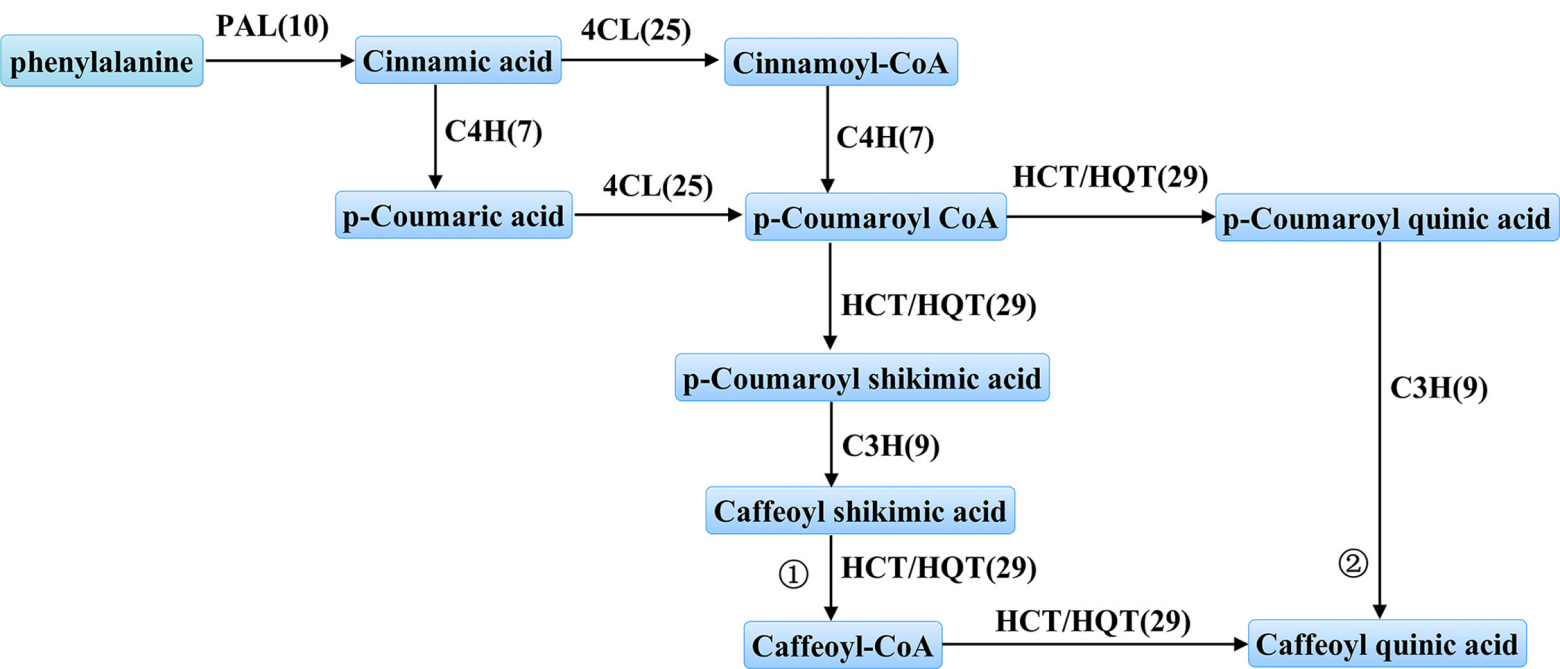
Proposed pathways for the biosynthesis of CGA in *Lonicera macranthoides*. The two different routes of CGA biosynthetic are labeled 1 and 2. Enzyme names are shown in the pictures. Each enzyme is annotated with the number of corresponding unigenes shown in parentheses. PAL, phenylalanine ammonia lyase; C4H, cinnamate 4-hydroxylase; 4CL, 4-hydroxycinnamoyl CoA ligase/4-coumarate-CoA ligase; HCT, hydroxycinnamoyl CoA shikimate/quinate hydroxycinnamoyltransferase; C3H, p-coumarate 39-hydroxlase; HQT, hydroxycinnamoyl CoA quinate hydroxycinnamoyl transferase.

*L*. *macranthoides* is a relative of *L*. *japonica*, and the CGA content in *L*. *macranthoides* was two-fold higher than of the content in *L*. *japonica* [41]. Correspondingly, transcriptome sequencing demonstrated that the number of genes involved in CGA synthesis in *L*. *macranthoides* was greater than the number in *L*. *japonica* (Table 2 and S3 Table). There are 10 *PAL*, 25 *4CL*, 9 *C3H* and 29 *HCT/HQT* genes in *L*. *macranthoides*, whereas there are 8 *PAL*, 21 *4CL*, 5 *C3H* and 3 *HCT/HQT* genes in *L*. *japonica* [29]. The phenylpropanoid biosynthesis pathway involved in CGA biosynthesis (ko00940) was identified using KEGG metabolic pathways. Moreover, candidate metabolic pathways for CGA biosynthesis were identified (Fig 3). Unlike the commonly recognized three pathways for CGA biosynthesis in *Lonicera japonica* or other species [29], only two biosynthesis pathways were identified in *L*. *macranthoides*. See panels 1 and 2 of Fig 3.

**Fig 4:**
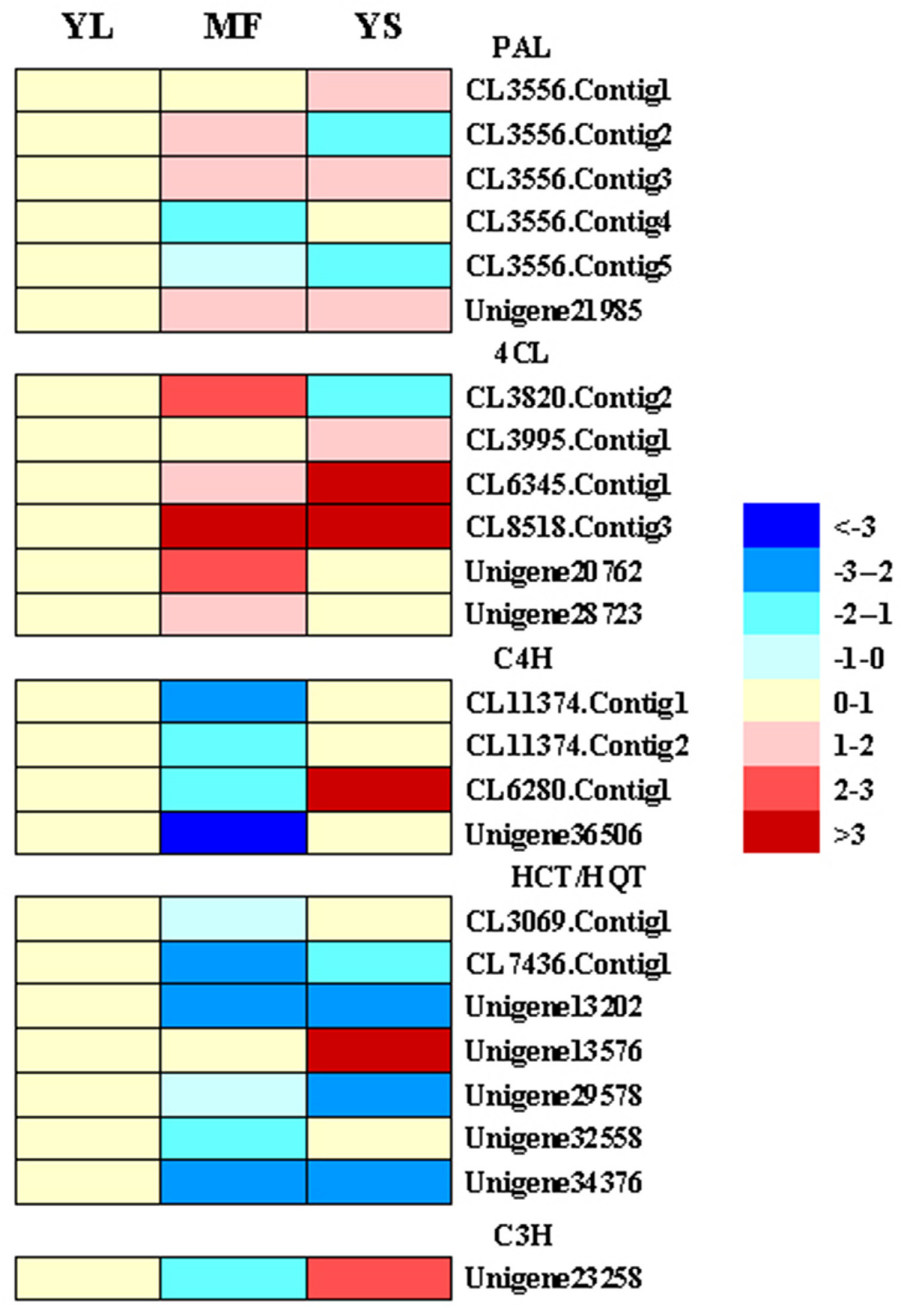
Cluster analysis of DEGs related to CGA biosynthesis.

RNA-Seq showed that some of the genes predicted to be involved in CGA synthesis were differentially expressed among YL, YS and MF in *L*. *macranthoides*. Using NCBI BLAST alignment, we identified genes encoding key enzymes in the CGA biosynthesis pathway (Fig 4). These genes included six *PAL*, six *4CL*, four *C4H*, one *C3H* and seven *HCT/HQT* genes. One *PAL* gene (CL3556.contig5) was down-regulated in YS and MF compared with YL, and three genes (CL3556.contig1, CL3556.contig3 and Unigene21985) were up-regulated. Moreover, the expression levels of CL3556.contig2 and 4 were up- and down-regulated respectively and down-regulated in MF respectively as compared to YL, whereas CL3556.contig2 expression was down-regulated in YS with respect to YL. The six *4CL* genes identified by RNA-Seq showed up-regulation in MF in comparison to YL. Compared to YL, except for CL3820.contig2, all *4CL* genes were up-regulated in YS. Four *C4H* genes were differentially expressed in the three tissues. With respect to YL, except for CL6280.contig1 showing up-regulation, no obvious change was observed in the other three genes in YS. Furthermore, all four *C4H* genes were down-regulated in MF. Seven *HCT/HQT* genes were differentially expressed in different tissues. The other six genes showed significantly lower expression in MF than in YL excepted for Unigene13576. Similarly, the genes including CL7436.contig1, Unigene13202, Unigene29578 and Unigene34376 were dramatically down-regulated in YS compared with YL. The expression of one *C3H* gene (Unigene23258) was down-regulated in MF compared with YL, while it was up-regulated in YS. These DEGs, especially those functioning downstream in the metabolic pathway, may provide valuable clues for elucidating the CGA biosynthesis pathway.

**Fig 5:**
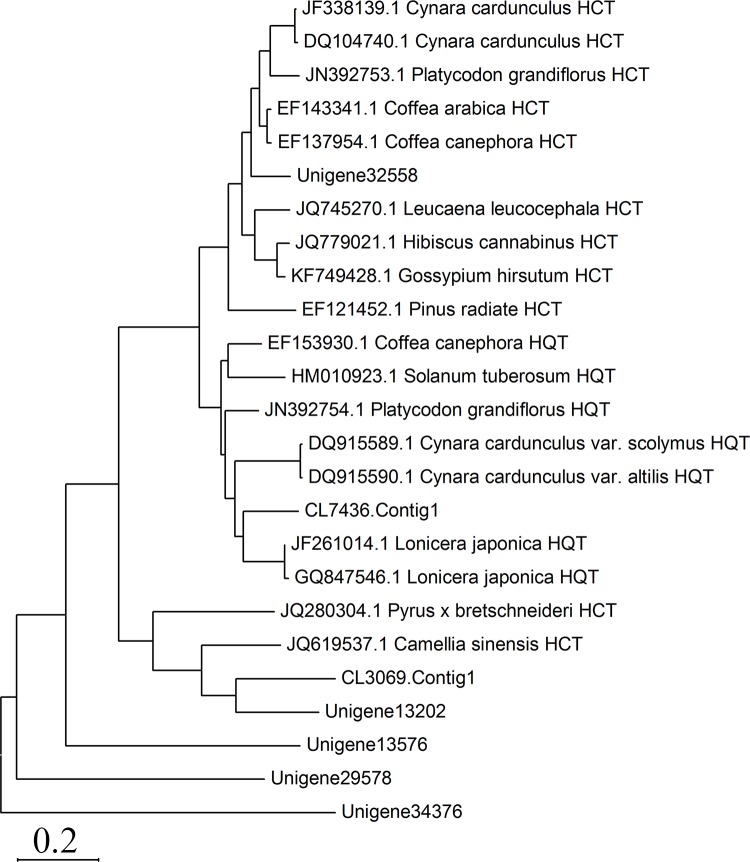
Protein sequence alignment of seven putative HQTs and HCTs in *Lonicera macranthoides* with 18 representative reference sequences in NCBI database. Neighbor-joining tree of HQTs and HCTs from *L*. *japonica* and other plants. The HQTs and HCTs used in phylogenetic analysis were retrieved from NCBI, including *Camellia sinensis* (JQ619537.1), *Coffea canephora* (EF137954.1, EF153930.1), *Coffea Arabica* (EF143341.1), *Cynara cardunculus* (JF338139.1, DQ104740.1), *Cynara cardunculus* var. altilis (DQ915590.1), *Cynara cardunculus* var. scolymus (DQ915589.1), *Gossypium hirsutum* (KF749428.1), *Hibiscus cannabinus* (JQ779021.1), *Leucaena leucocephala* (JQ745270.1), *Lonicera japonica* (JF261014.1, GQ847546.1), *Pinus radiate* (EF121452.1), *Platycodon grandiflorus* (JN392753.1, JN392754.1), *Pyrus x bretschneideri* (JQ280304.1), *Solanum tuberosum* (HM010923.1).

Many studies have shown that *HCT* and *HQT* are key genes involved in CGA biosynthesis in plants [24,25,26]. To further explore the evolutionary relationships of *HCT/HQT* genes between *L*. *macranthoides* and other species, a phylogenetic tree was generated based on seven differentially expressed *HCT/HQT* genes and 18 sequences obtained from NCBI. Unigene32558 was found to share a relatively high sequence homology with the *HCT* genes in *Cynara cardunculus* (JF338139.1, DQ104740.1), *Platycodon grandiflorus* (JN392753.1), *Coffea canephora* (EF137954.1, EF153930.1) and *Coffea Arabica* (EF143341.1) (Fig 5). Similarly, among the seven *HCT/HQT* unigenes, CL7436.congtig1 is classified into same subcluster with *HQT* sequences and shares the highest sequence homology with *HQT* in *L*. *japonica* (JF261014.1, GQ847546.1). It is noteworthy that the CL3069.contig1 and Unigene13202 share relatively high sequence homology with the HCT sequences in *Pyrus bretschneideri* (JQ280304.1) and *Camellia sinensis* (JQ619537.1), whereas Unigene13576, Unigene29578 and Unigene34376 did not show high sequence homology with *HCT/HQT* genes in other species.

### Validation of RNA-Seq Data by qRT-PCR

**Fig 6:**
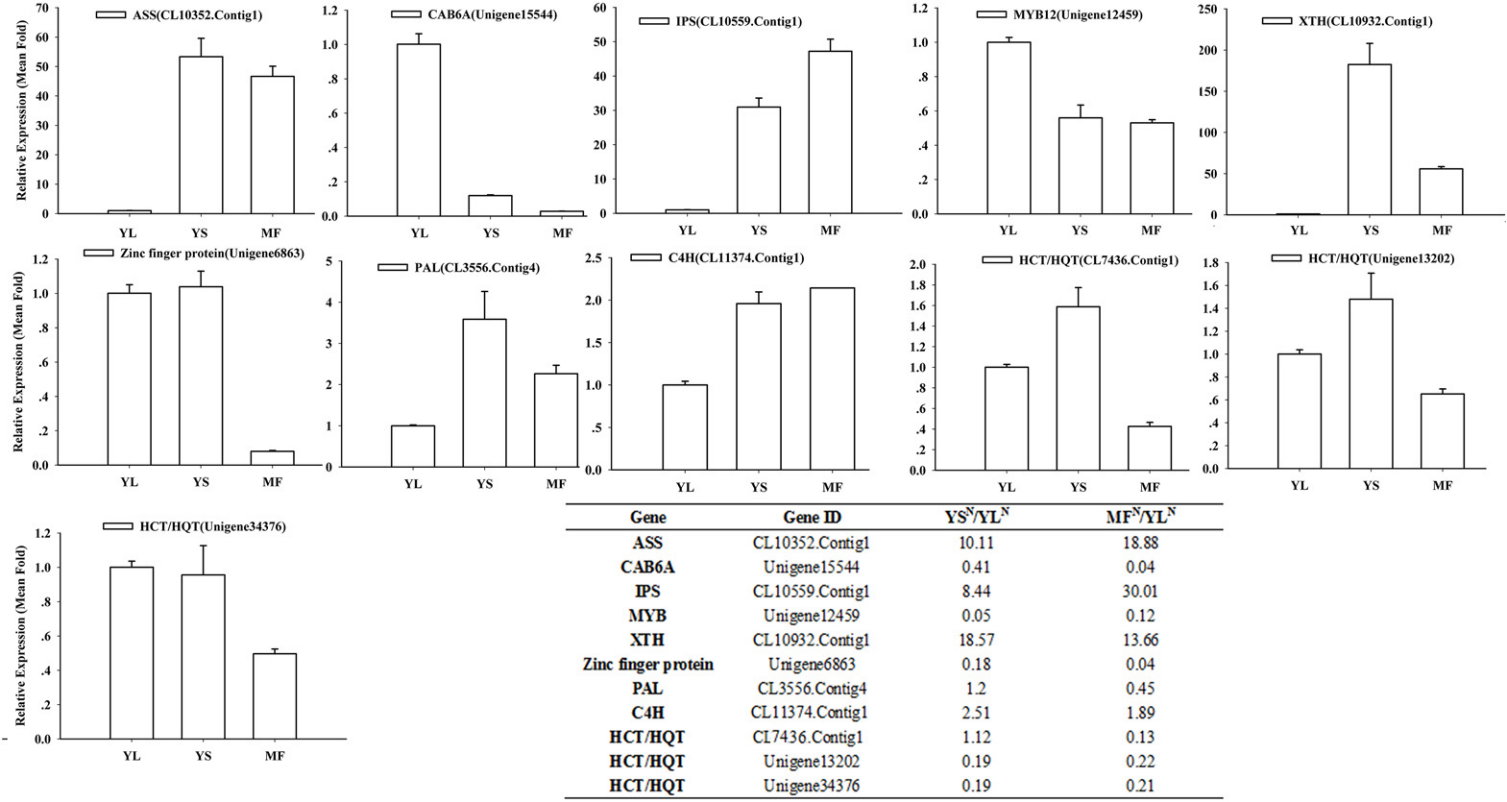
Expression patterns of selected genes identified by RNA-Seq were validated by qRT-PCR. Results of qPCR were presented in histograms, and results of RNA-Seq were listed in the embedded table.

To validate the accuracy of the RNA-Seq data, qPCR was performed on five candidate genes associated with CGA biosynthesis (*PAL*: CL3556.contig4; *C4H*: CL11374.Contig2; *HQT/HCT*: CL7436.Contig1, Unigene13202_LmYL, and Unigene34376_LmYL) and six genes randomly selected from the expression profile data (*Zinc*: Unigene6863_LmYL; *CAB*: Unigene15544_LmYL; *IPS*: CL10559.Contig1_LmYL; *ASS*: CL10352.contig1_LmYL; *XTH*: CL10932.contig1_LmYL; *MYB*: Unigene12459_LmYL). All of these genes were differentially expressed among the three different tissues, and the data were consistent with the RNA-Seq data (Fig 6). Therefore, our results provide reliable transcriptome and expression profile data for further investigations of key genes involved in the CGA biosynthesis pathway in *L*. *macranthoides*.

## Discussion

*L*. *macranthoides*, commonly known as mountain honeysuckle, is an important Chinese herb medicine that is native to the southwest and central provinces of China. The CGA content in *L*. *macranthoides* is higher than that in traditional honeysuckle in Northern China (*Lonicera japonica*) [42]. However, the molecular mechanism underlying CGA biosynthesis in *L*. *macranthoides* remains unclear. Several studies have focused on the transcriptome and expression profile of genes related to CGA synthesis in *L*. *japonica* [13,29,30]. However, few described similar data CGA biosynthesis in *L*. *macranthoides* [13,26]. Moreover, there are no reports on the metabolic pathway and transcriptome underlying the biosynthesis of CGA, a core secondary metabolite in *L*. *macranthoides*. We used the Illumina HiSeq™ 2000 platform to investigate gene expression profiles and the key pathways for CGA biosynthesis in *L*. *macranthoides*. CGA content differs significantly in different tissues of *L*. *macranthoides*. The RNA from MF, YS and YL samples of *L*. *macranthoides* was mixed in equal amounts for transcriptome sequencing. A total of 76,453 unigenes were obtained. Based on these data, RNA-Seq analysis was performed on the three different tissues and DEGs were identified.

CGA content in *Lonicera* plants is determined by multiple factors including developmental stage and growth environment [43,44], treatment methods [45] and tissues region [46,47]. Our study found that CGA content varied in different stages of development (data not shown). Moreover, we found significant differences in the CGA content of different tissues of *L*. *macranthoides*, which was highest in YL and lowest in MF (Fig 1D). This result differs from the previous reports that CGA content in the flowers was higher than that in the leaves of *L*. *japonica* [13], which could be due to the differences in the growth environment or the development stage of the collected samples. CGAs are important phenolic acids produced in many plant species via secondary metabolism. Currently, three main pathways, which involve many important enzymes, have been identified in CGA biosynthesis pathways in plants [29]. Studies in *L*. *japonica*, coffee, artichoke and other plants have revealed that *PAL*, *C4H* and *4CL* are the common enzymes in the upstream CGA metabolic pathway [24,29,48]. The present study identified two possible pathways for CGA biosynthesis and candidate genes related to CGA biosynthesis in *L*. *macranthoides* (Fig 3). These two pathways were identified in previous studies in *L*. *japonica*, coffee, artichoke, tobacco, tomato and other plants [20,24]. The third pathway, in which caffeoyl glucoside serves as an activated intermediate, has only been reported in sweet potato [49]. And, DEGs related to CGA biosynthesis were identified, including six *PAL*, four *C4H*, six *C4L*, one *C3H* and seven *HQT/HCT* genes. Among them, the expression level of a *PAL* gene (CL3556.contig4) corresponded well with CGA content in different tissues (Fig 1 and Fig 6). Similarly, its orthologous gene *PAL1* (79% identity) in Coffea canephora was identified to be in high expression at the small green stage, which correlated with the high production of CGA during the SG stage [50]. These results indicate the involvement of *PAL* in CGA accumulation. We also found that the transcripts of three *C4H* genes (CL11374.contig1, CL11374.contig2 and Unigene36506) were positively correlated with the CGA content, indicating their important role in CGA biosynthesis. On the contrary, a previous study in *L*. *japonica* displayed that *LjC4H2* is critical gene that regulate CGA content within the organs, not *LjC4H1* which showed highest identity to the *C4H* genes (CL11374.contig1 and CL11374.contig2) in *L*. *macranthoides* identified in our study [13]. Besides,the correlations (either positive or negative) between CGA content and gene expressions of the remaining *PAL*, *C4H* and *4CL* genes in different tissues were very weak (Fig 4). These unigenes in the upstream of CGA biosynthesis pathways may be shared by the phenylpropanoid pathway and other metabolic pathways [29] and are not the key rate-limiting enzymes in *L*. *macranthoides* [23]. It is also likely that different homologous genes encoding the same enzyme may differ in function. Future transgenic experiments targeting each transcript may further explore the key genes involved in CGA biosynthesis in *L*. *macranthoides*.

*HQT* and *HCT* are homologous genes involved in the phenylpropanoid biosynthetic pathway. *HCT* can use both shikimate and quinate as substrates, whereas *HQT* only uses quinate. *HCT* is a key enzyme in the phenylpropanoid and lignin biosynthetic pathways, which was supported by the facts that silence of *HCT* gene led to significant changes in lignin content of *Arabidopsis*[37,51]. The role of *HCT* in CGA biosynthesis has been demonstrated in tobacco, tomato [20] and artichoke [48,52]. However, *HQT* is only involved in CGA biosynthesis. Two isoforms of *HQT*, *HQT1* and *HQT2*, contribute to CGA synthesis in artichoke [24]. In the present study, 7 unigenes encoding *HQT/HCT* showed organ-differential expression, and the transcripts of 6 *HQT/HCT* genes were correlated with CGA content in different tissues (Fig 4), suggesting their key roles in CGA biosynthesis in *L*. *macranthoides*. Among them, unigene32558 shares a relatively high sequence homology with the *HCT* genes in artichoke and coffee (Fig 5), which were considered to be involved in the synthesis of CGA [48,52,53]. CL7436.congtig1 shares the highest homology with the *HQT* gene in *L*. *japonica* (Fig 5). However, it’s a controversial issue about the role of this gene in CGA biosynthesis. Peng et al. [26] showed that tissue distribution of *HQT* was in accordance with the pattern of CGA content, indicating its essential role in CGA biosynthesis. Whereas, Yuan et al. [13] found that *LjHQTs* did not show significant organ preferential expression and may not be the critical genes regulating CGA content. And our results strongly support the forward view. In addition, CL3069.contig1 and Unigene13202 share relatively high sequence similarity with *HCT/HQT* of some species and are thus candidate *HCT/HQT* genes (Fig 5). However, the functions of these genes need to be verified in future studies.

## Conclusion

In summary, we sequenced the transcriptome of *L*. *macranthoides* and investigated the relationship between CGA content and genes associated with CGA biosynthesis. Transcriptome analysis revealed that 3767 unigenes classified into 33 pathways are involved in the biosynthesis of secondary metabolites. These data provide genomic resources for further study of the molecular mechanism of secondary metabolism in *L*. *macranthoides*. Furthermore, we identified 447 genes associated with pathways for phenylpropanoid biosynthesis, and we found two candidate pathways for CGA biosynthesis in *L*. *macranthoides*. Several genes, including one *PAL*, three *C4H* and four *HCT/HQT* genes, showed correlation with CGA content, suggesting their key role in CGA biosynthesis.These results characterize a potential molecular regulatory mechanism for CGA biosynthesis, and in doing so provide a wealth of candidate genes for future studies to engineer improvement of *L*. *macranthoides*.

## Supporting Information

S1 Fig General information on the transcriptome. (JPG)

S2 Fig Classification of total raw reads in three different tissues. After filtering the only adaptor sequences, containing N sequences and low quality sequences, the three RNA-Seq libraries still generated over 3.5 million clean reads in each library and the percentage of clean reads among raw tags in each library over 99.4%.
(JPG)

S1 File Evaluation of transcriptome sequencing quality in different tissues. A, Classfication of raw reads. B, Distribution analysis of the RNA-Seq tags in the three libraries.
(JPG)

S2 File The differentially expressed genes between MF, YL and YS libraries in *Lonicera macranthoides* Hand.-Mazz. A, The differentially expressed genes between MF and YL libraries; B, The differentially expressed genes between MF and YS libraries; C, The differentially expressed genes between YS and YL libraries. (XLSX)

S1 Table Primer sequences used for qRT-PCR. (XLSX)

S2 Table Summary for the *Lonicera macranthoides* transcriptome. (DOC)

S3 Table Genes associated with CGA synthesis identified by RNA-Seq in different tissues from *Lonicera macranthoides* Hand.-Mazz. (XLSX)
