# Supplementary figures and images for: Transcriptome Analysis Reveals the Mechanism Underlying the Production of a High Quantity of Chlorogenic Acid in Young Leaves of Lonicera macranthoides Hand.-Mazz
Source: PLoS One. 2015 Sep 18;10(9):e0137212. doi: 10.1371/journal.pone.0137212 (PMC4575056; doi:10.1371/journal.pone.0137212)

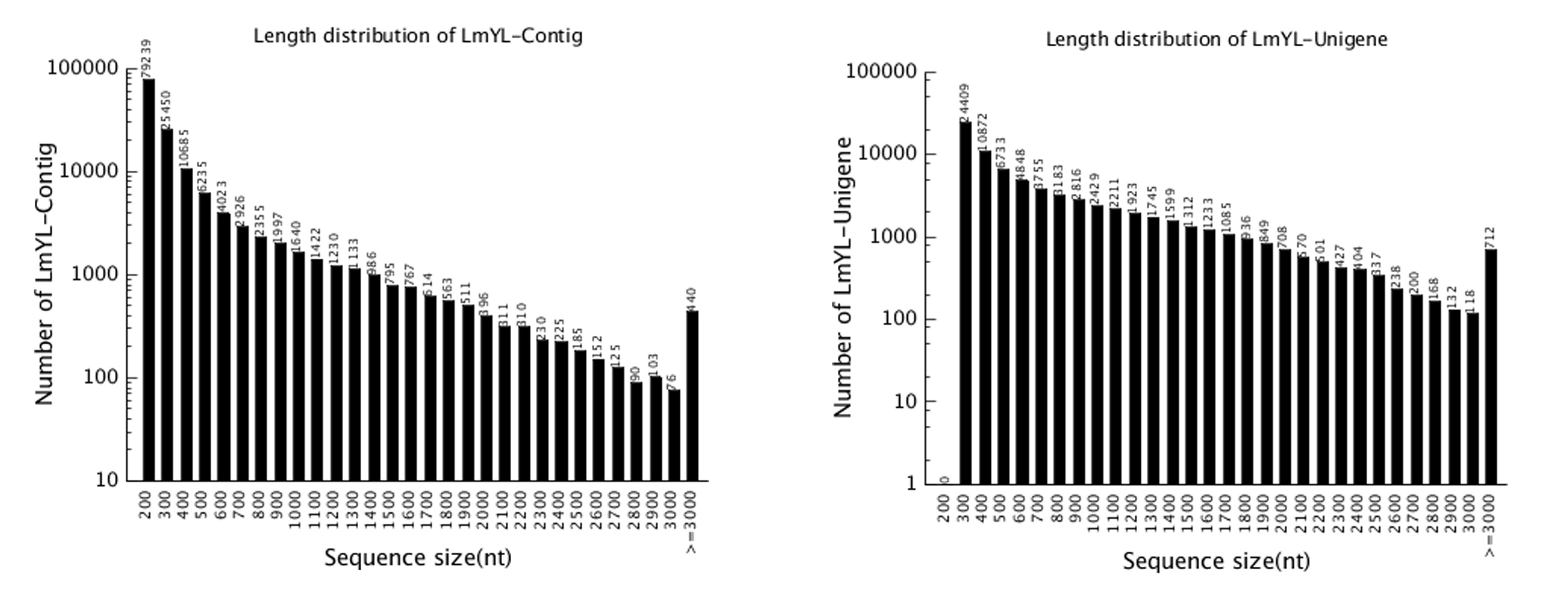

Supplement: S1 Fig — (JPG) [file pone.0137212.s001.jpg]

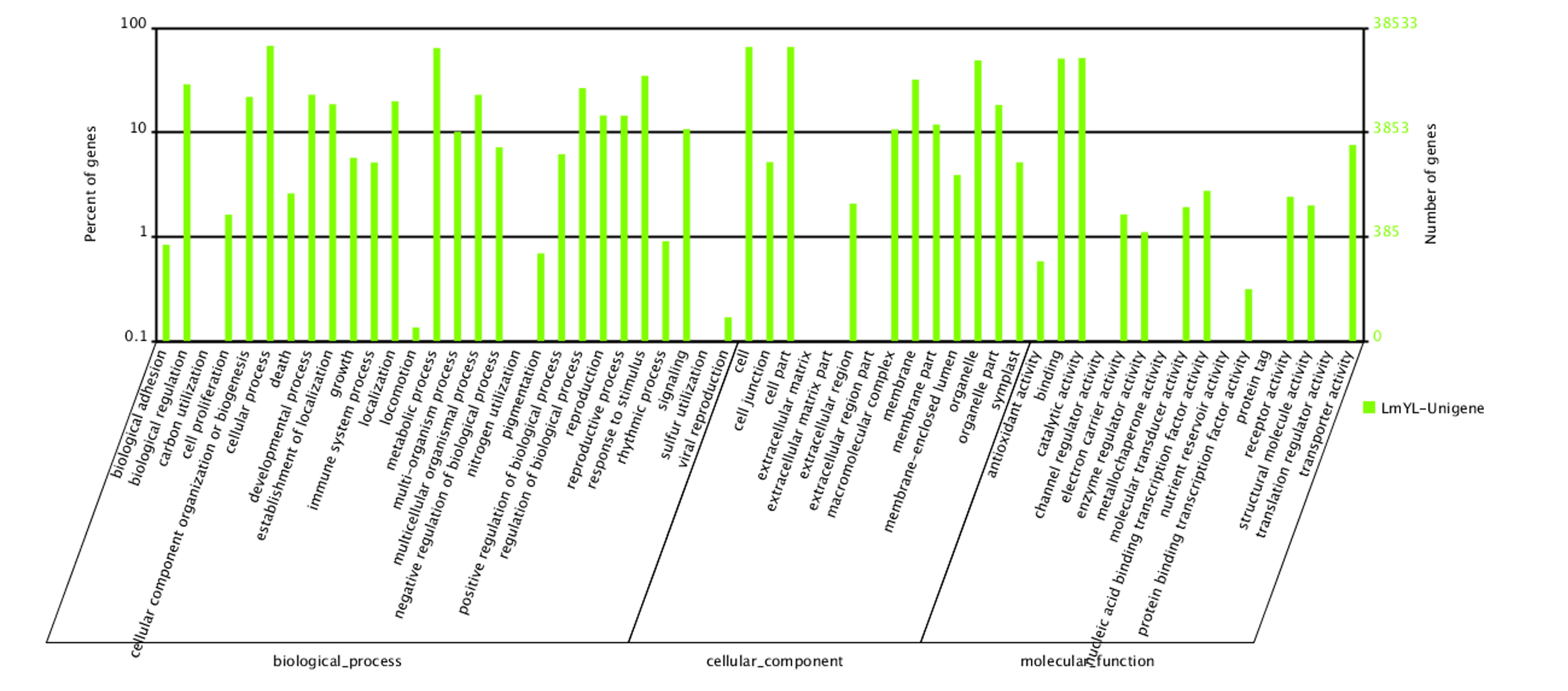

Supplement: S2 Fig — After filtering the only adaptor sequences, containing N sequences and low quality sequences, the three RNA-Seq libraries still generated over 3.5 million clean reads in each library and the percentage of clean reads among raw tags in each library over 99.4%. (JPG) [file pone.0137212.s002.jpg]

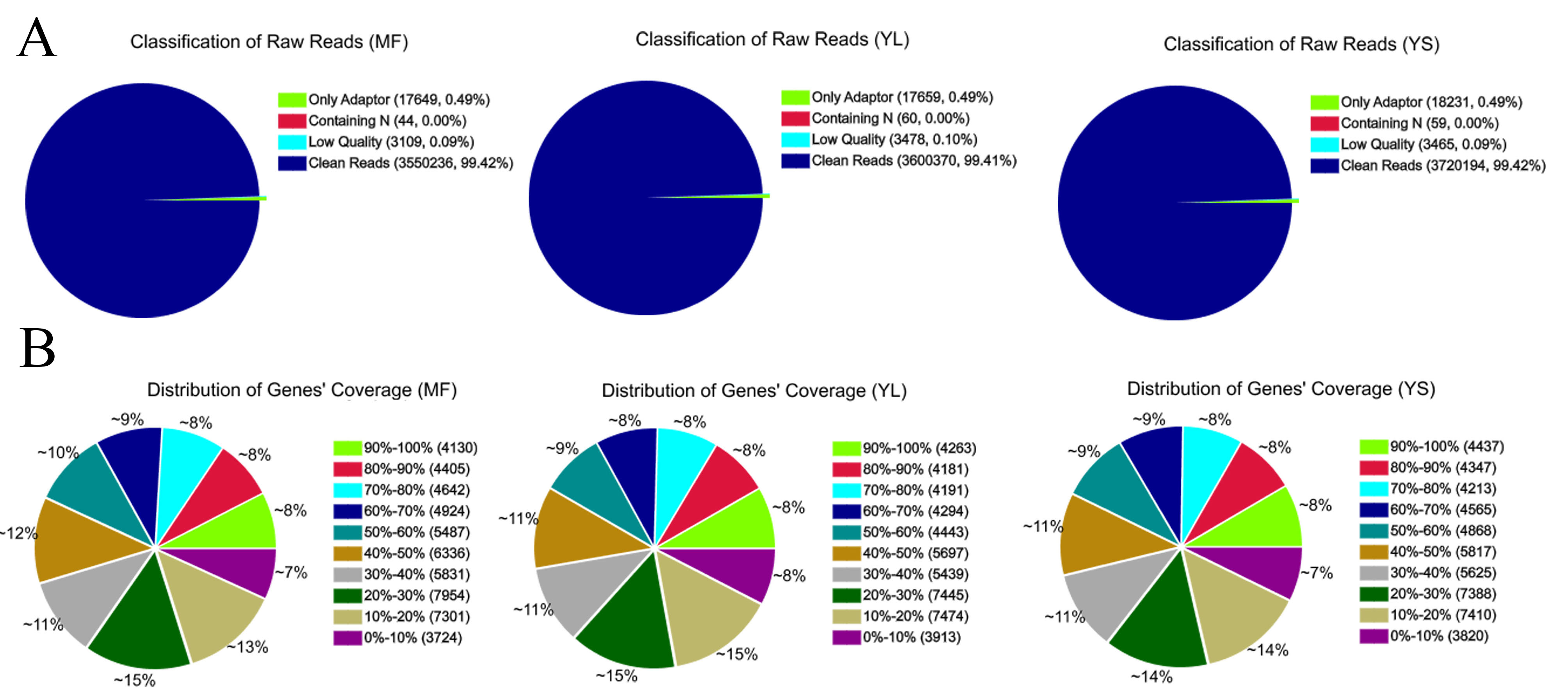

Supplement: S1 File — A, Classfication of raw reads. B, Distribution analysis of the RNA-Seq tags in the three libraries. (JPG) [file pone.0137212.s003.jpg]
