# Supplementary material for: Transcriptome Analysis Reveals the Mechanism Underlying the Production of a High Quantity of Chlorogenic Acid in Young Leaves of Lonicera macranthoides Hand.-Mazz
Source: PLoS One. 2015 Sep 18;10(9):e0137212. doi: 10.1371/journal.pone.0137212 (PMC4575056; doi:10.1371/journal.pone.0137212)
Supplement: S2 Table — (DOC) [file pone.0137212.s006.doc]

**SI Table 2 Summary for the** ***Lonicera macranthoides* transcriptome**

| Total number of raw reads 60287590  Total number of clean reads 53533014  Total number of clean nucleotides (bp) 4817971260  Average Read Length (bp) 90  Total number of contigs 145214  Total length of contigs (bp) 49696505  Mean length of contigs (bp) 342  Total number of unigenes 76453  Total length of unigenes (bp) 53749724  Mean length of unigenes 703  N50 1088 |
| --- |
